# Supplementary figures and images for: Trade Integration and Trade Imbalances in the European Union: A Network Perspective
Source: PLoS One. 2014 Jan 22;9(1):e83448. doi: 10.1371/journal.pone.0083448 (PMC3898927; doi:10.1371/journal.pone.0083448)

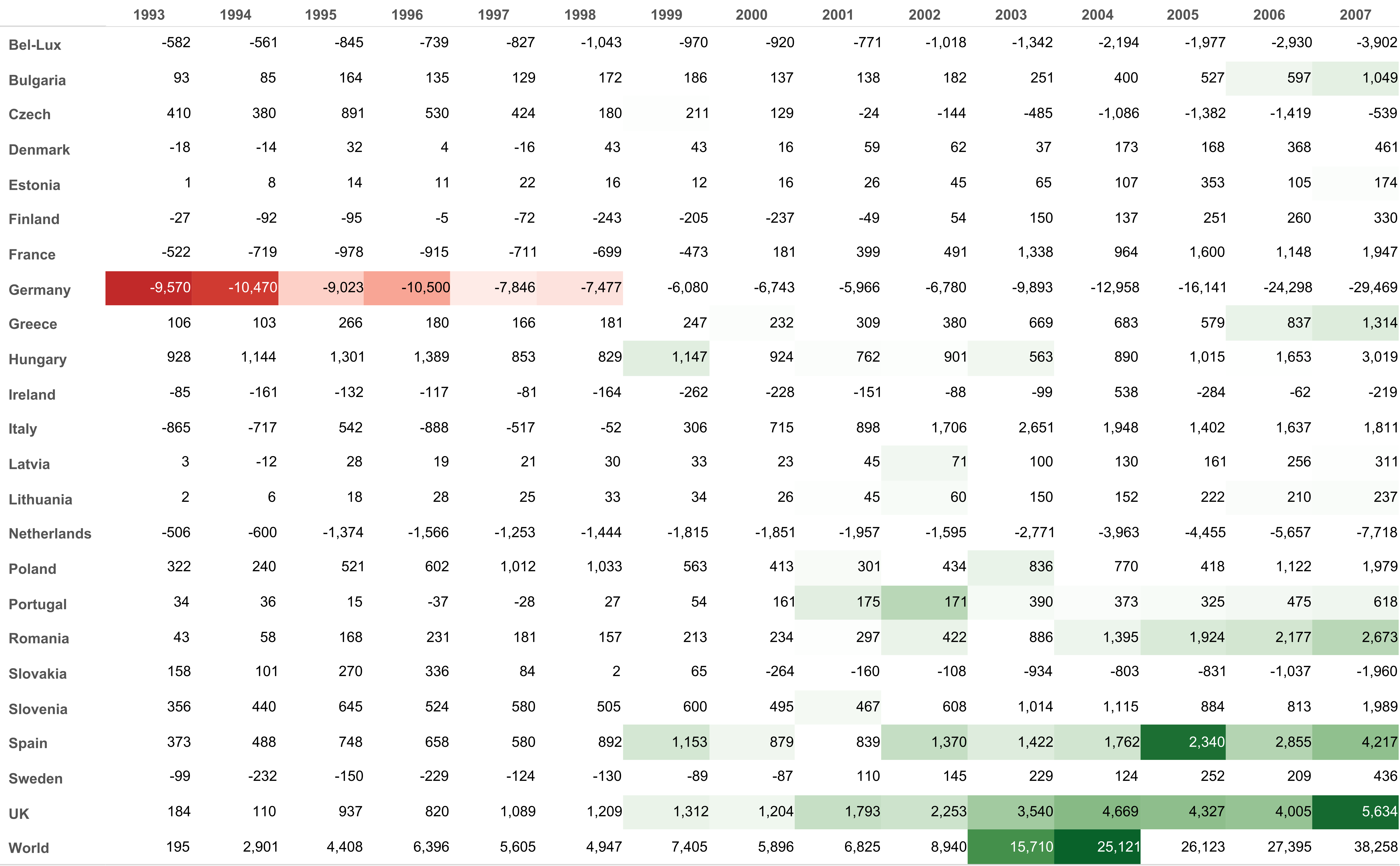

Supplement: Figure S1 — Evolution of the direct and indirect measures of trade imbalances for Austria. The figures in each cell correspond to direct trade surpluses (+) or deficits (−) of Austria toward countries listed on the rows. The colors correspond to the indirect measures of trade imbalances, as computed by the Flow Decomposition Method, with ultimate surpluses in green and ultimate deficits in red. (TIFF) [file pone.0083448.s001.tiff]

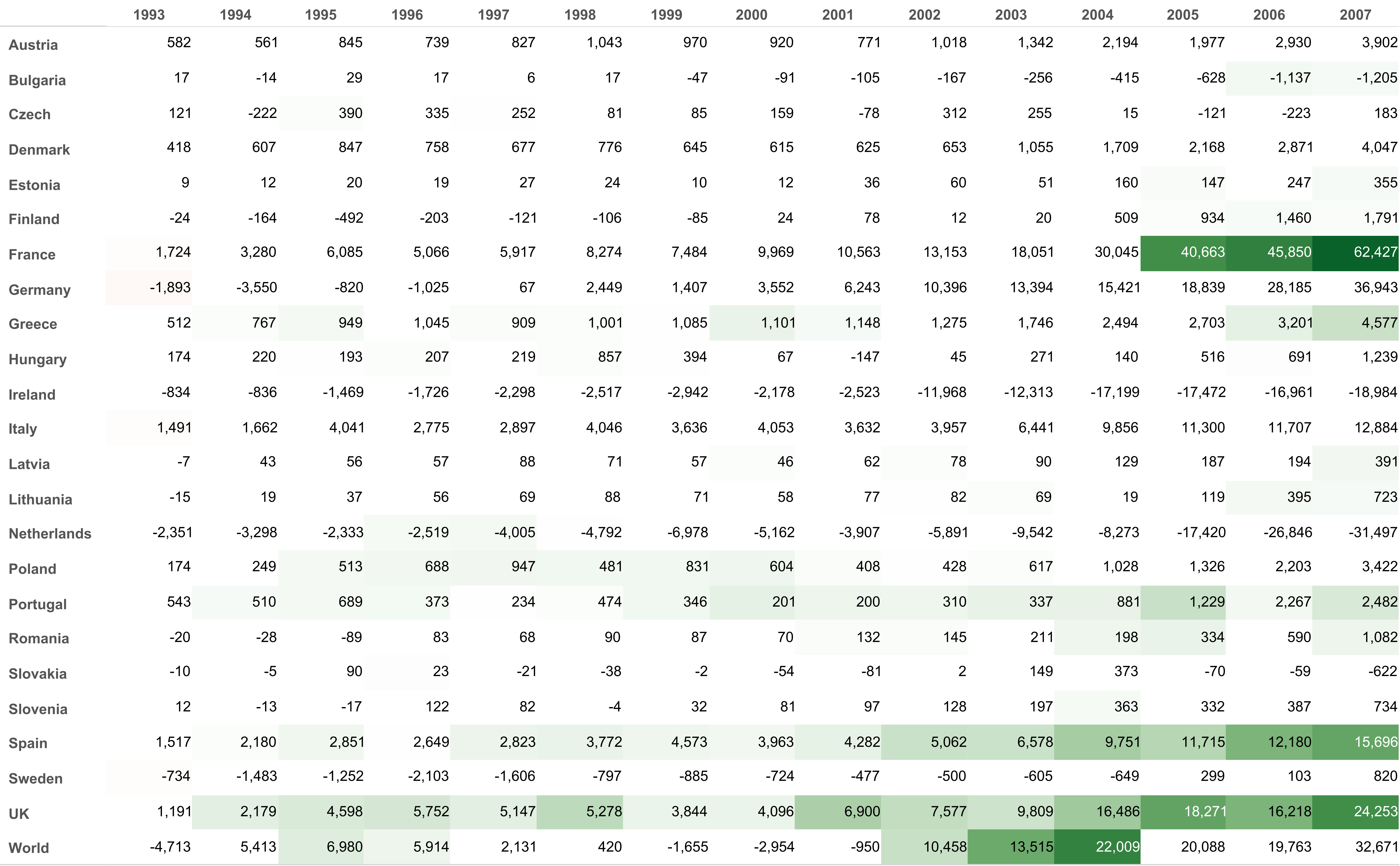

Supplement: Figure S2 — Evolution of the direct and indirect measures of trade imbalances for Belgium and Luxembourg. The figures in each cell correspond to direct trade surpluses (+) or deficits (−) of Belgium and Luxembourg toward countries listed on the rows. The colors correspond to the indirect measures of trade imbalances, as computed by the Flow Decomposition Method, with ultimate surpluses in green and ultimate deficits in red. (TIFF) [file pone.0083448.s002.tiff]

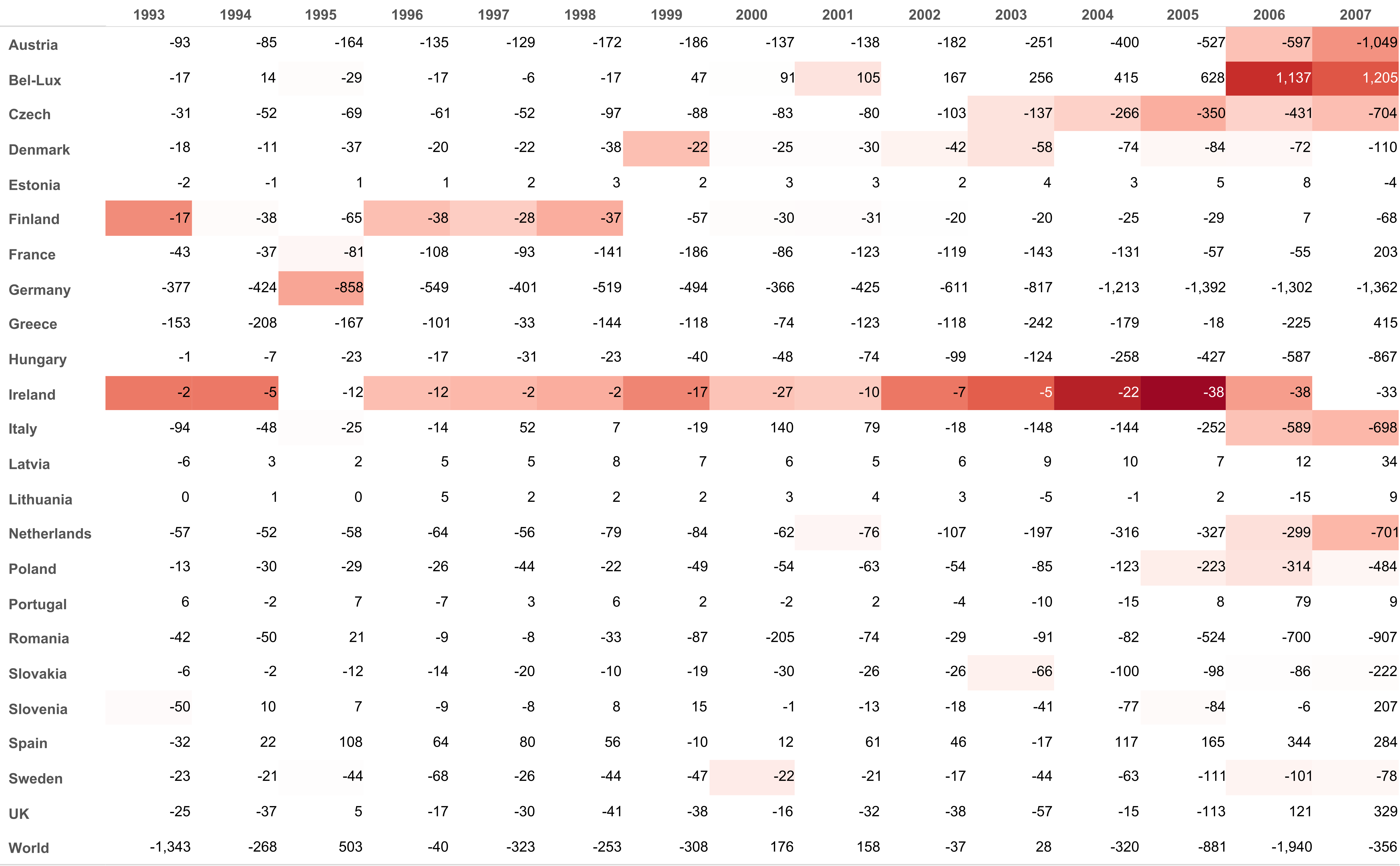

Supplement: Figure S3 — Evolution of the direct and indirect measures of trade imbalances for Bulgaria. The figures in each cell correspond to direct trade surpluses (+) or deficits (−) of Bulgaria toward countries listed on the rows. The colors correspond to the indirect measures of trade imbalances, as computed by the Flow Decomposition Method, with ultimate surpluses in green and ultimate deficits in red. (TIFF) [file pone.0083448.s003.tiff]

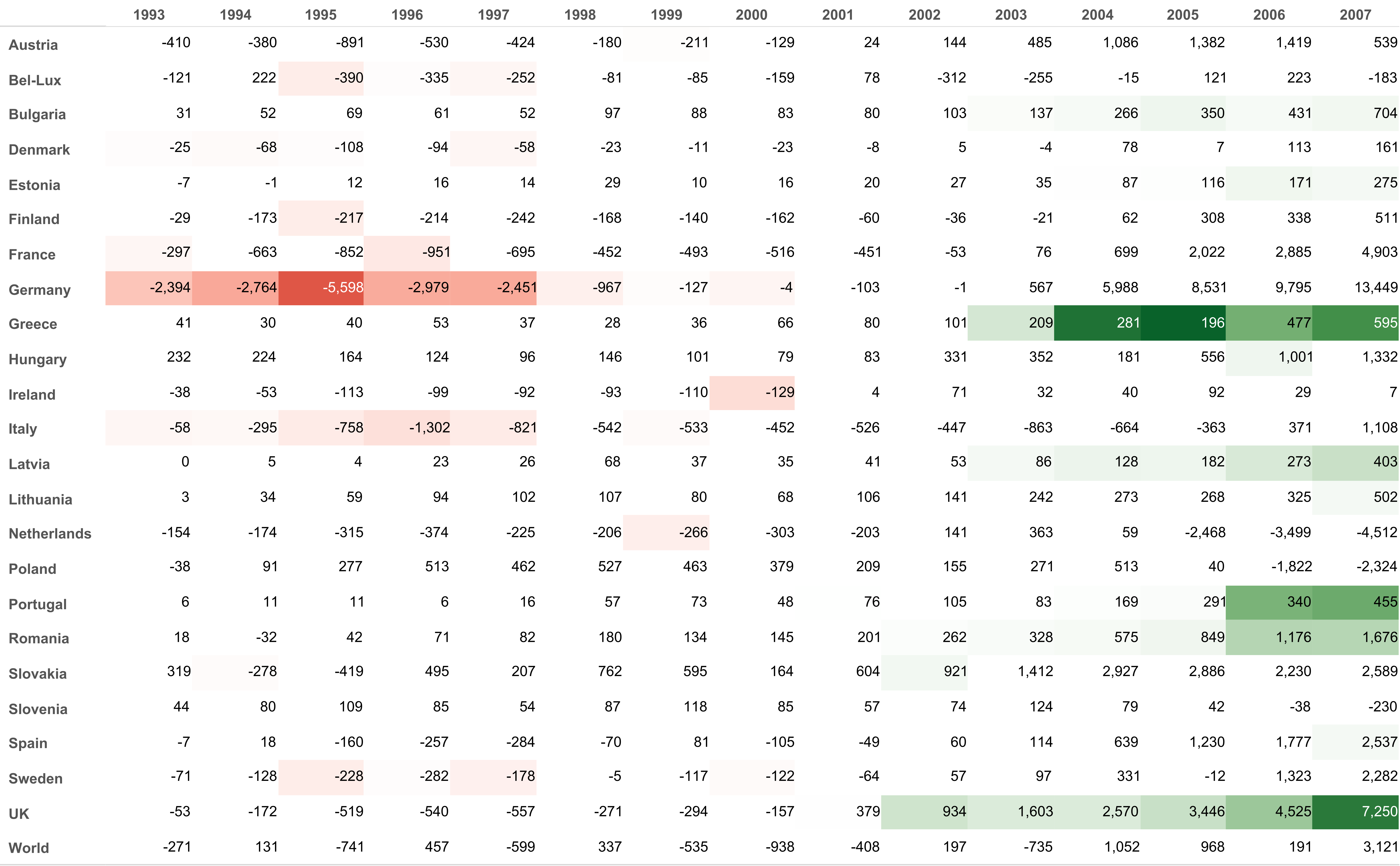

Supplement: Figure S4 — Evolution of the direct and indirect measures of trade imbalances for Czech Republic. The figures in each cell correspond to direct trade surpluses (+) or deficits (−) of Czech Republic toward countries listed on the rows. The colors correspond to the indirect measures of trade imbalances, as computed by the Flow Decomposition Method, with ultimate surpluses in green and ultimate deficits in red. (TIFF) [file pone.0083448.s004.tiff]

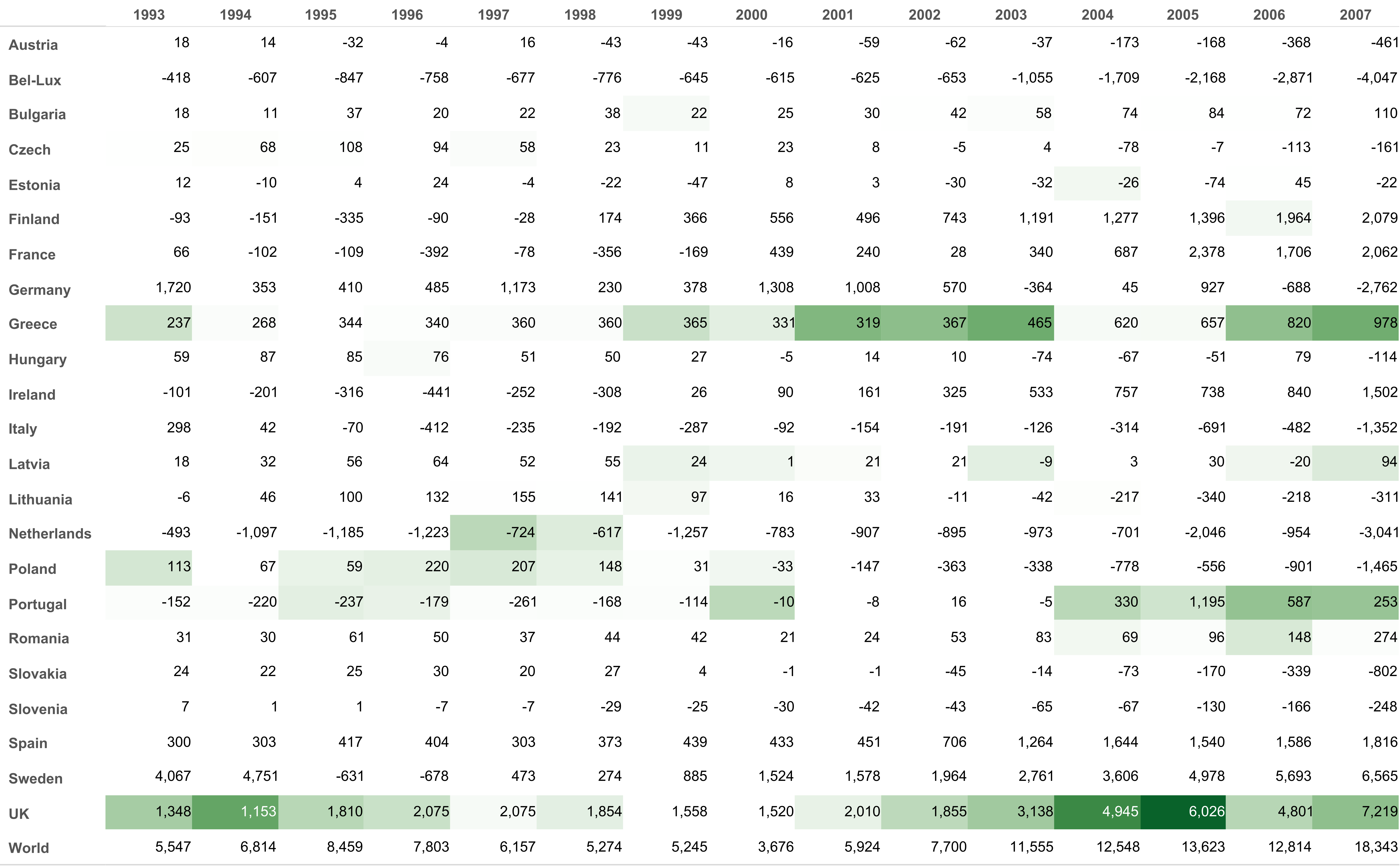

Supplement: Figure S5 — Evolution of the direct and indirect measures of trade imbalances for Demnark. The figures in each cell correspond to direct trade surpluses (+) or deficits (−) of Demnark toward countries listed on the rows. The colors correspond to the indirect measures of trade imbalances, as computed by the Flow Decomposition Method, with ultimate surpluses in green and ultimate deficits in red. (TIFF) [file pone.0083448.s005.tiff]

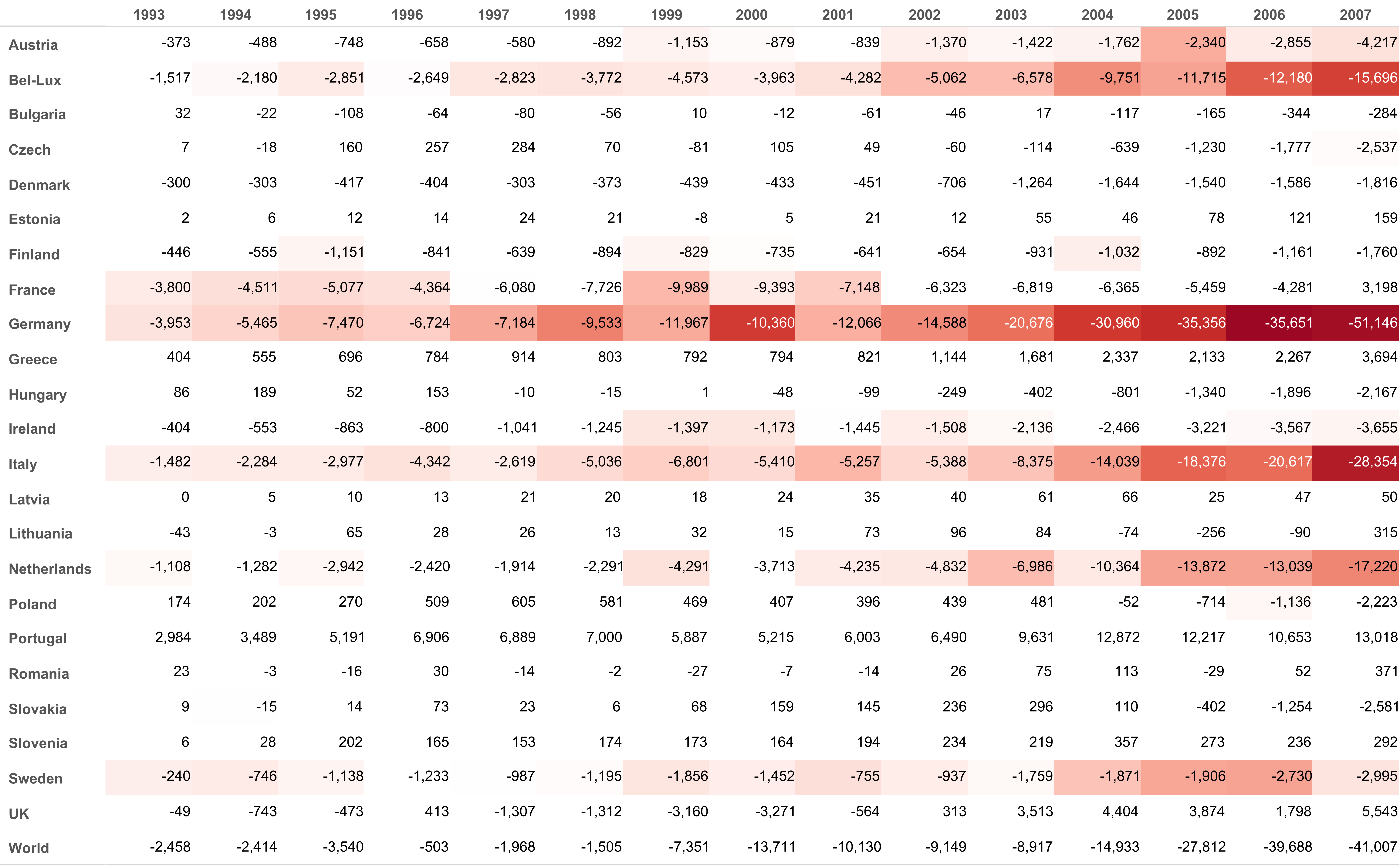

Supplement: Figure S6 — Evolution of the direct and indirect measures of trade imbalances for Spain. The figures in each cell correspond to direct trade surpluses (+) or deficits (−) of Spain toward countries listed on the rows. The colors correspond to the indirect measures of trade imbalances, as computed by the Flow Decomposition Method, with ultimate surpluses in green and ultimate deficits in red. (TIFF) [file pone.0083448.s006.tiff]

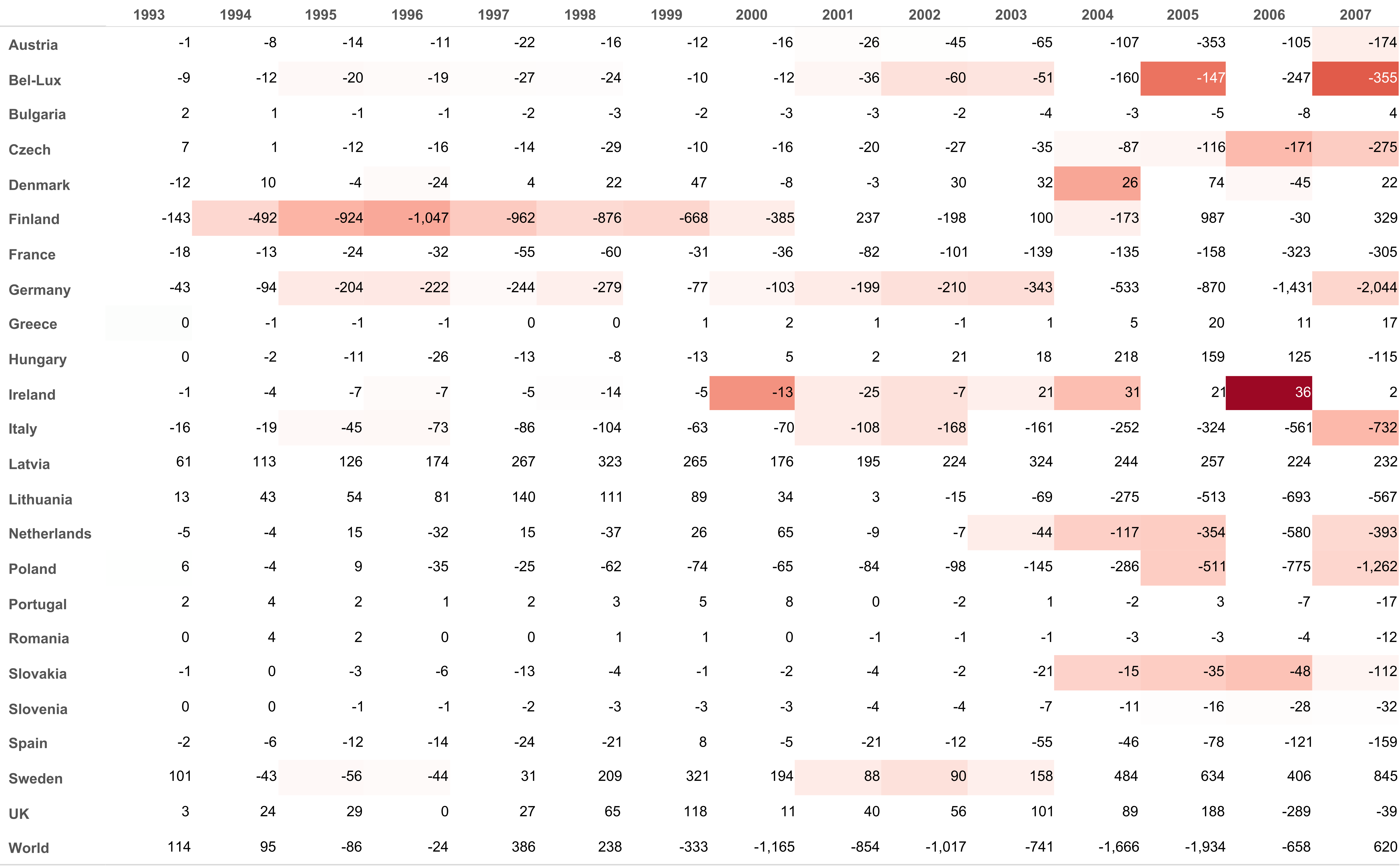

Supplement: Figure S7 — Evolution of the direct and indirect measures of trade imbalances for Estonia. The figures in each cell correspond to direct trade surpluses (+) or deficits (−) of Estonia toward countries listed on the rows. The colors correspond to the indirect measures of trade imbalances, as computed by the Flow Decomposition Method, with ultimate surpluses in green and ultimate deficits in red. (TIFF) [file pone.0083448.s007.tiff]

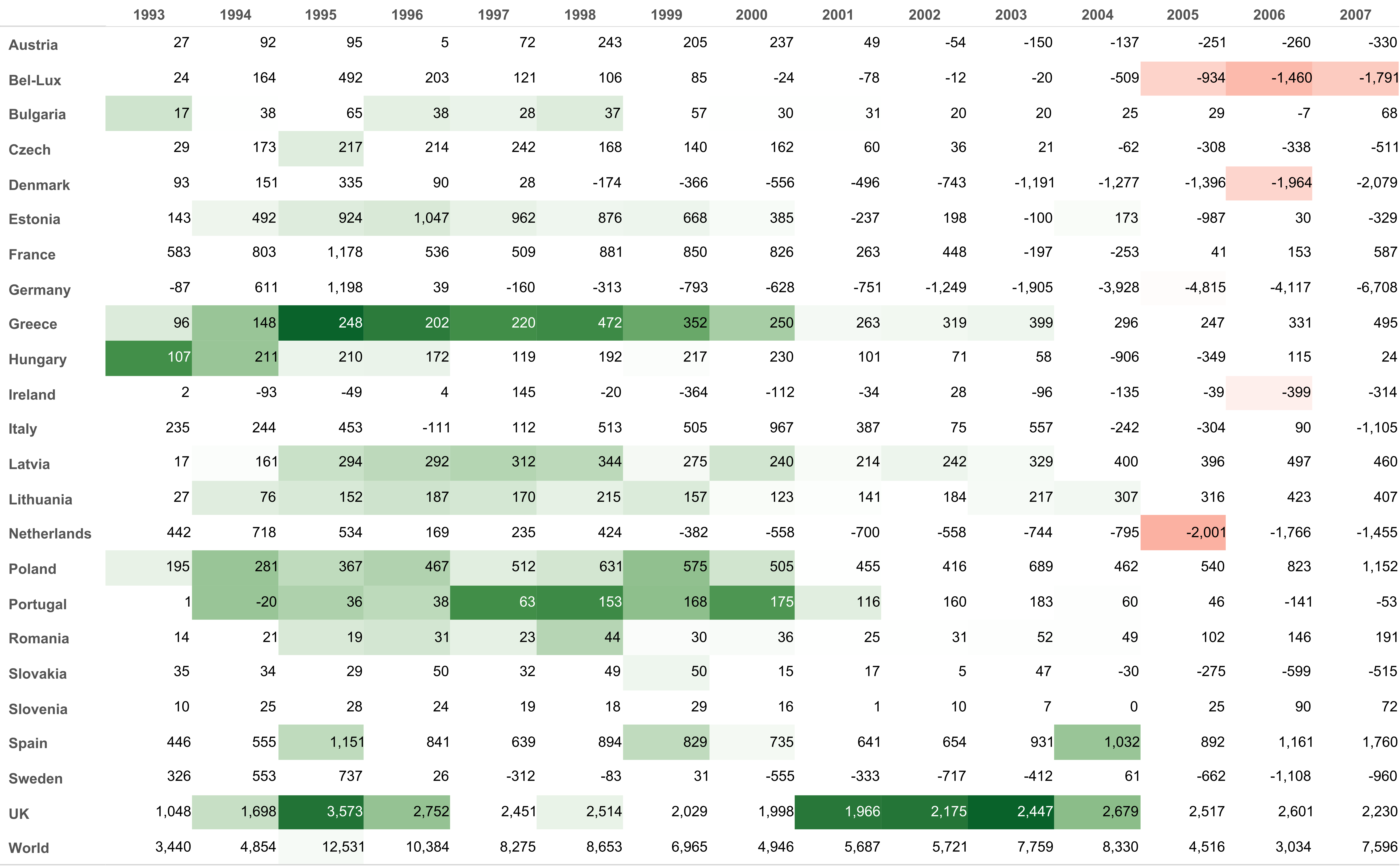

Supplement: Figure S8 — Evolution of the direct and indirect measures of trade imbalances for Finland. The figures in each cell correspond to direct trade surpluses (+) or deficits (−) of Finland toward countries listed on the rows. The colors correspond to the indirect measures of trade imbalances, as computed by the Flow Decomposition Method, with ultimate surpluses in green and ultimate deficits in red. (TIFF) [file pone.0083448.s008.tiff]

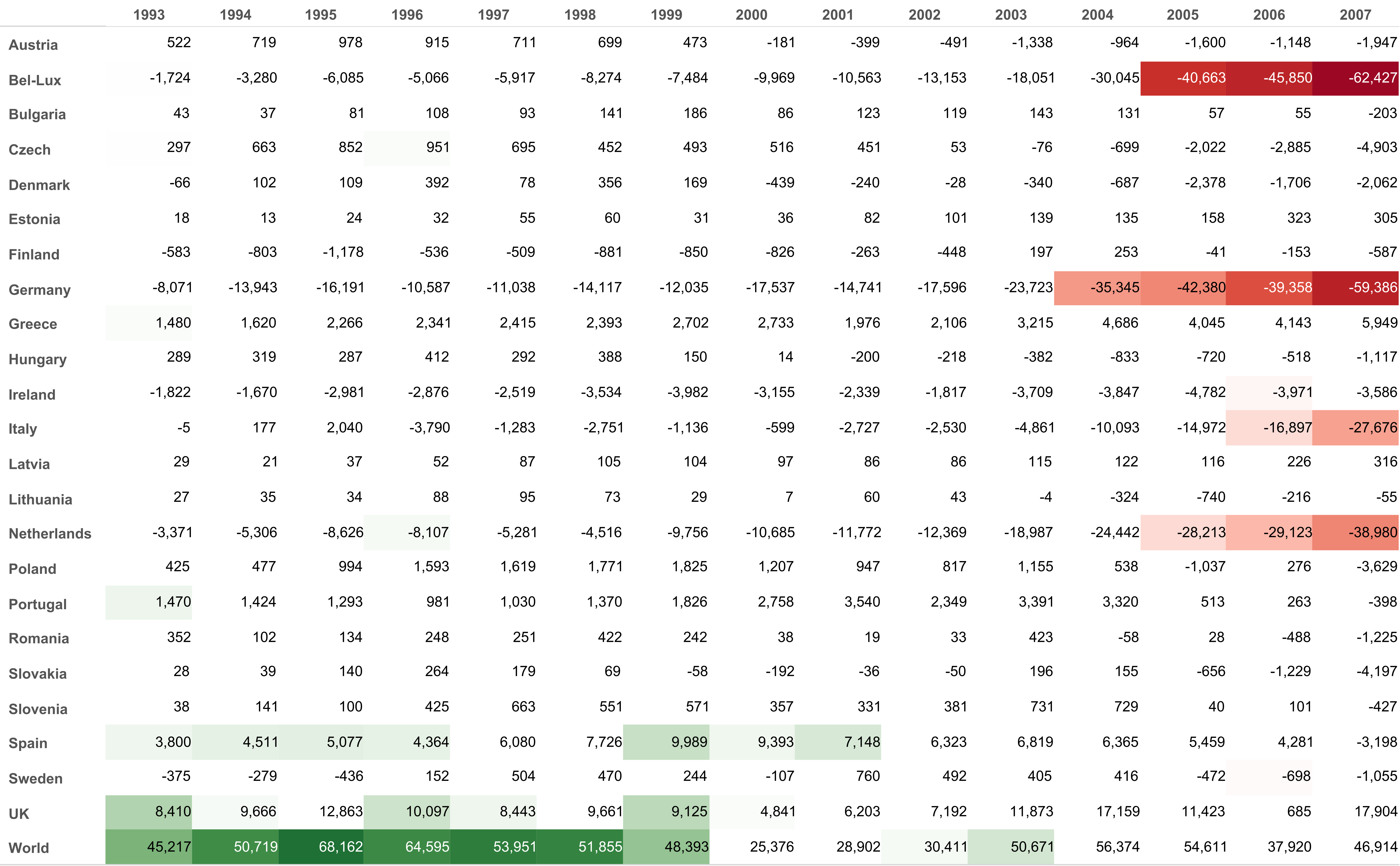

Supplement: Figure S9 — Evolution of the direct and indirect measures of trade imbalances for France. The figures in each cell correspond to direct trade surpluses (+) or deficits (−) of France toward countries listed on the rows. The colors correspond to the indirect measures of trade imbalances, as computed by the Flow Decomposition Method, with ultimate surpluses in green and ultimate deficits in red. (TIFF) [file pone.0083448.s009.tiff]

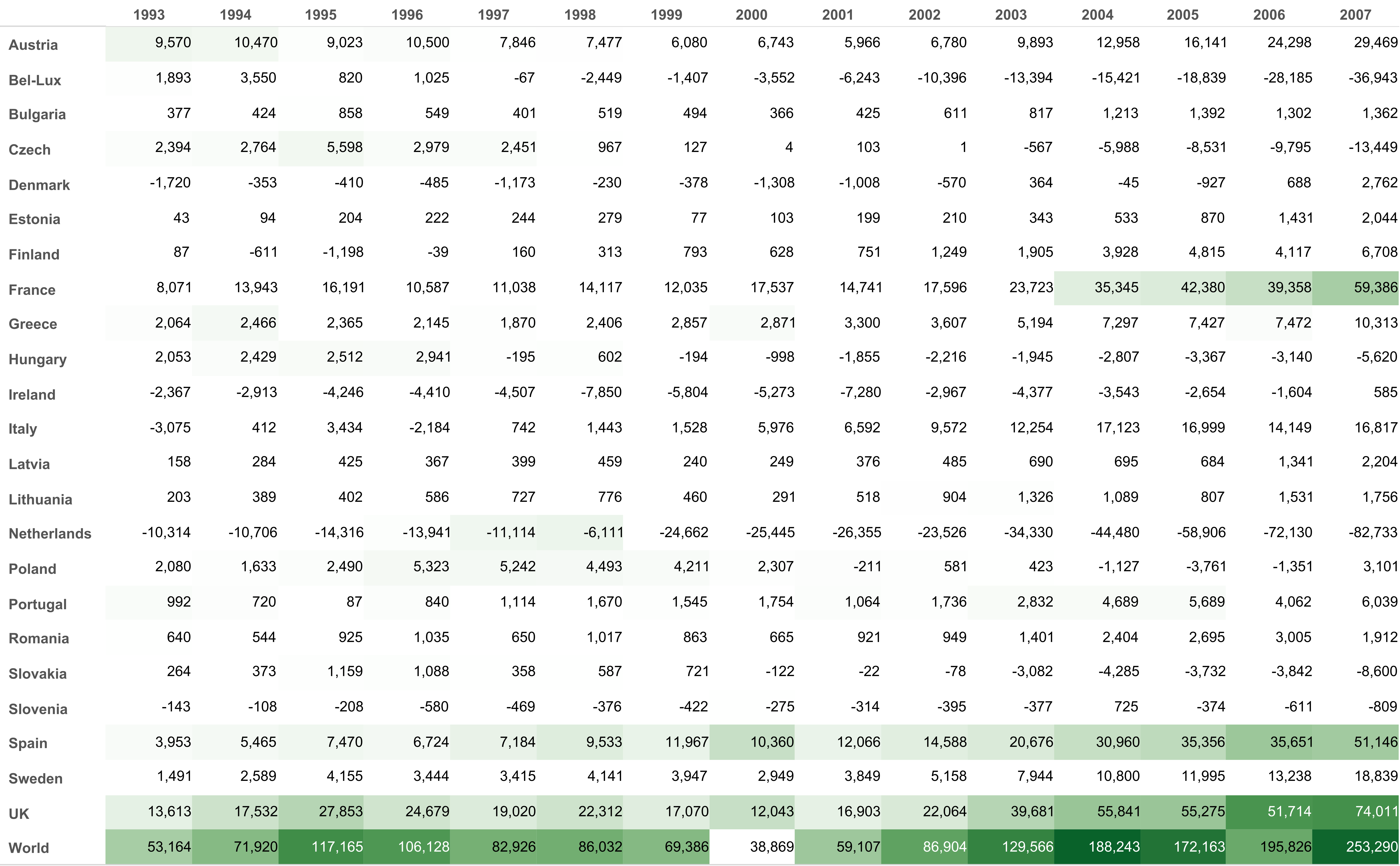

Supplement: Figure S10 — Evolution of the direct and indirect measures of trade imbalances for Germany. The figures in each cell correspond to direct trade surpluses (+) or deficits (−) of Germany toward countries listed on the rows. The colors correspond to the indirect measures of trade imbalances, as computed by the Flow Decomposition Method, with ultimate surpluses in green and ultimate deficits in red. (TIFF) [file pone.0083448.s010.tiff]

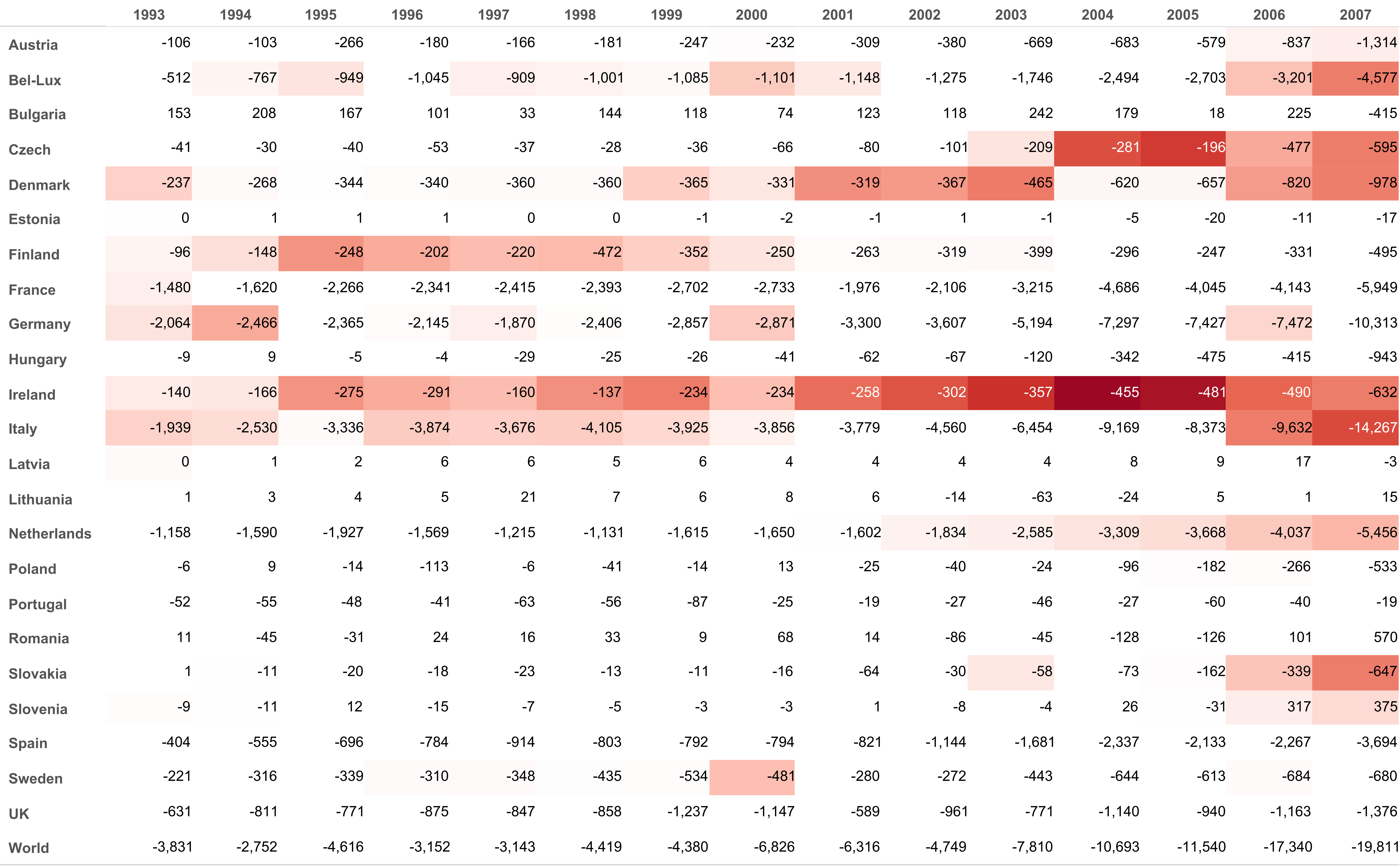

Supplement: Figure S11 — Evolution of the direct and indirect measures of trade imbalances for Greece. The figures in each cell correspond to direct trade surpluses (+) or deficits (−) of Greece toward countries listed on the rows. The colors correspond to the indirect measures of trade imbalances, as computed by the Flow Decomposition Method, with ultimate surpluses in green and ultimate deficits in red. (TIFF) [file pone.0083448.s011.tiff]

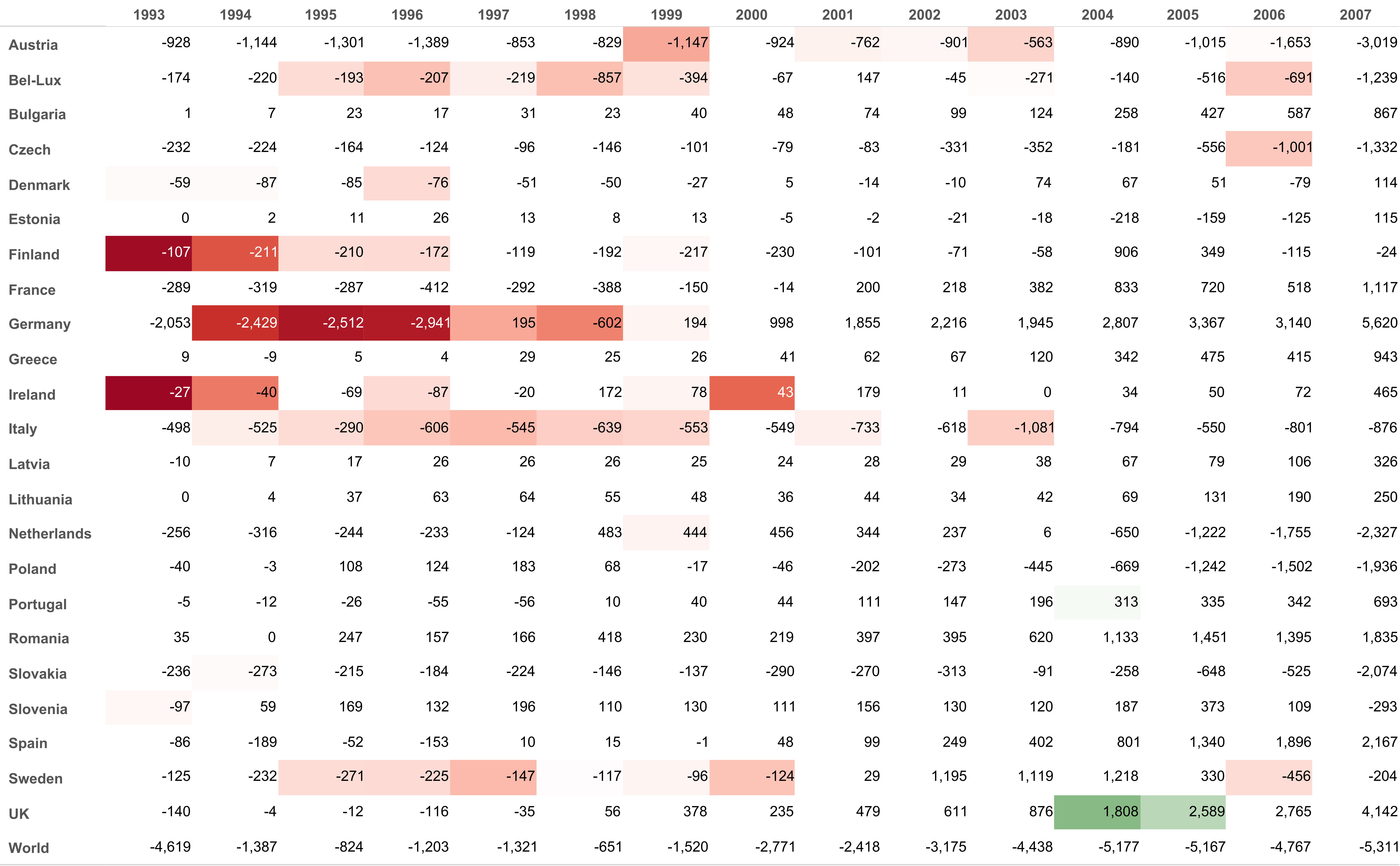

Supplement: Figure S12 — Evolution of the direct and indirect measures of trade imbalances for Hungary. The figures in each cell correspond to direct trade surpluses (+) or deficits (−) of Hungary toward countries listed on the rows. The colors correspond to the indirect measures of trade imbalances, as computed by the Flow Decomposition Method, with ultimate surpluses in green and ultimate deficits in red. (TIFF) [file pone.0083448.s012.tiff]

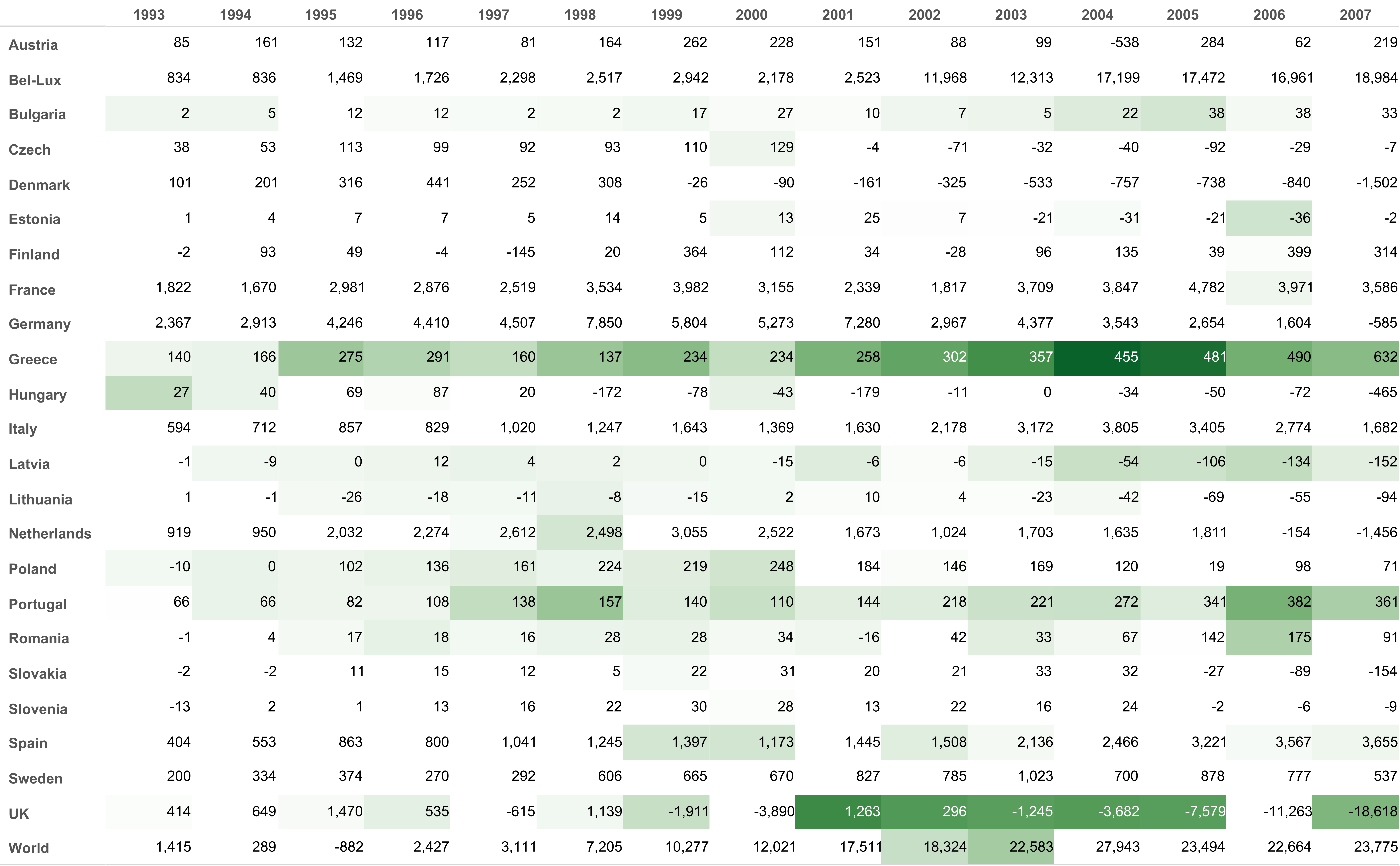

Supplement: Figure S13 — Evolution of the direct and indirect measures of trade imbalances for Ireland. The figures in each cell correspond to direct trade surpluses (+) or deficits (−) of Ireland toward countries listed on the rows. The colors correspond to the indirect measures of trade imbalances, as computed by the Flow Decomposition Method, with ultimate surpluses in green and ultimate deficits in red. (TIFF) [file pone.0083448.s013.tiff]

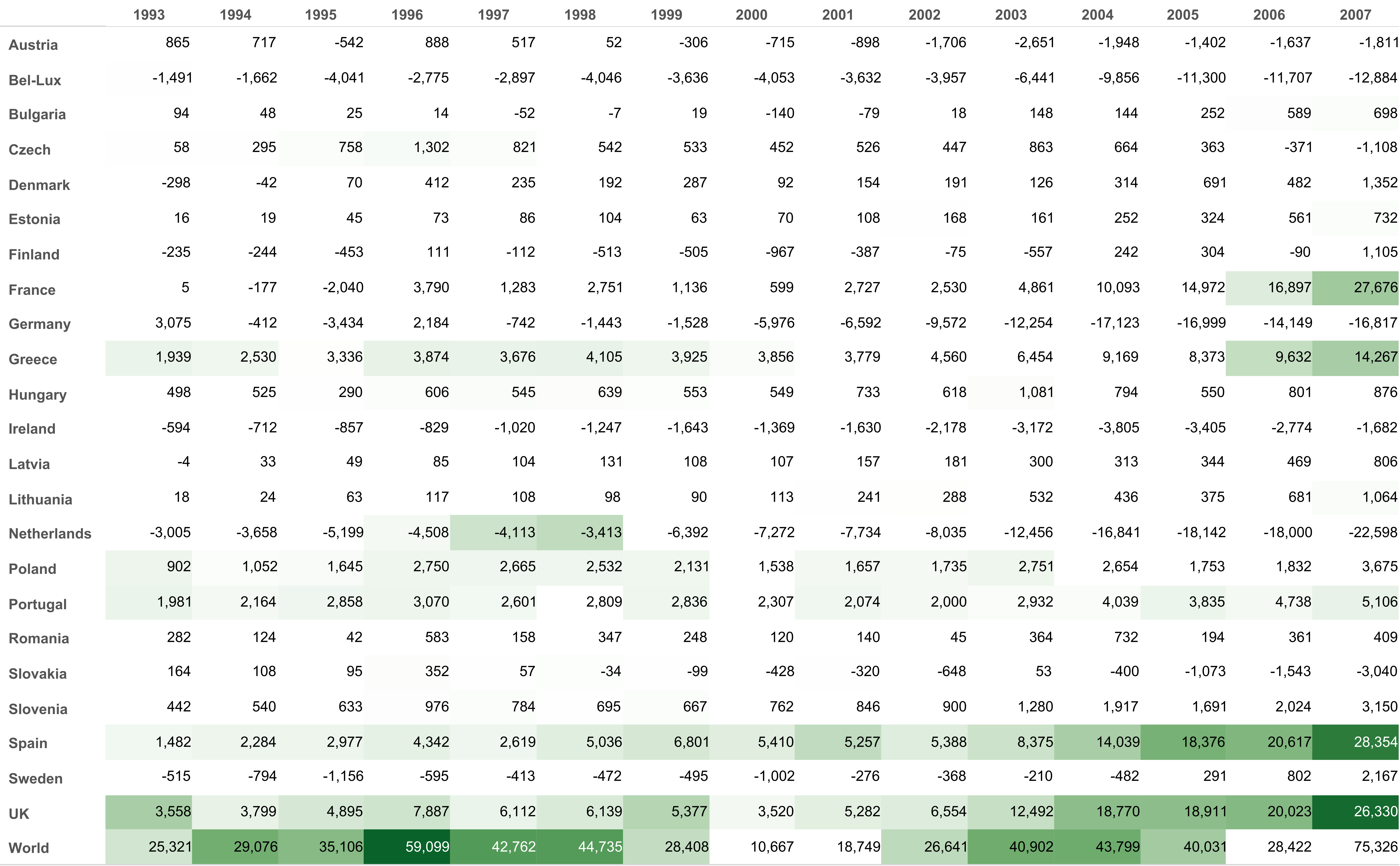

Supplement: Figure S14 — Evolution of the direct and indirect measures of trade imbalances for Italy. The figures in each cell correspond to direct trade surpluses (+) or deficits (−) of Italy toward countries listed on the rows. The colors correspond to the indirect measures of trade imbalances, as computed by the Flow Decomposition Method, with ultimate surpluses in green and ultimate deficits in red. (TIFF) [file pone.0083448.s014.tiff]

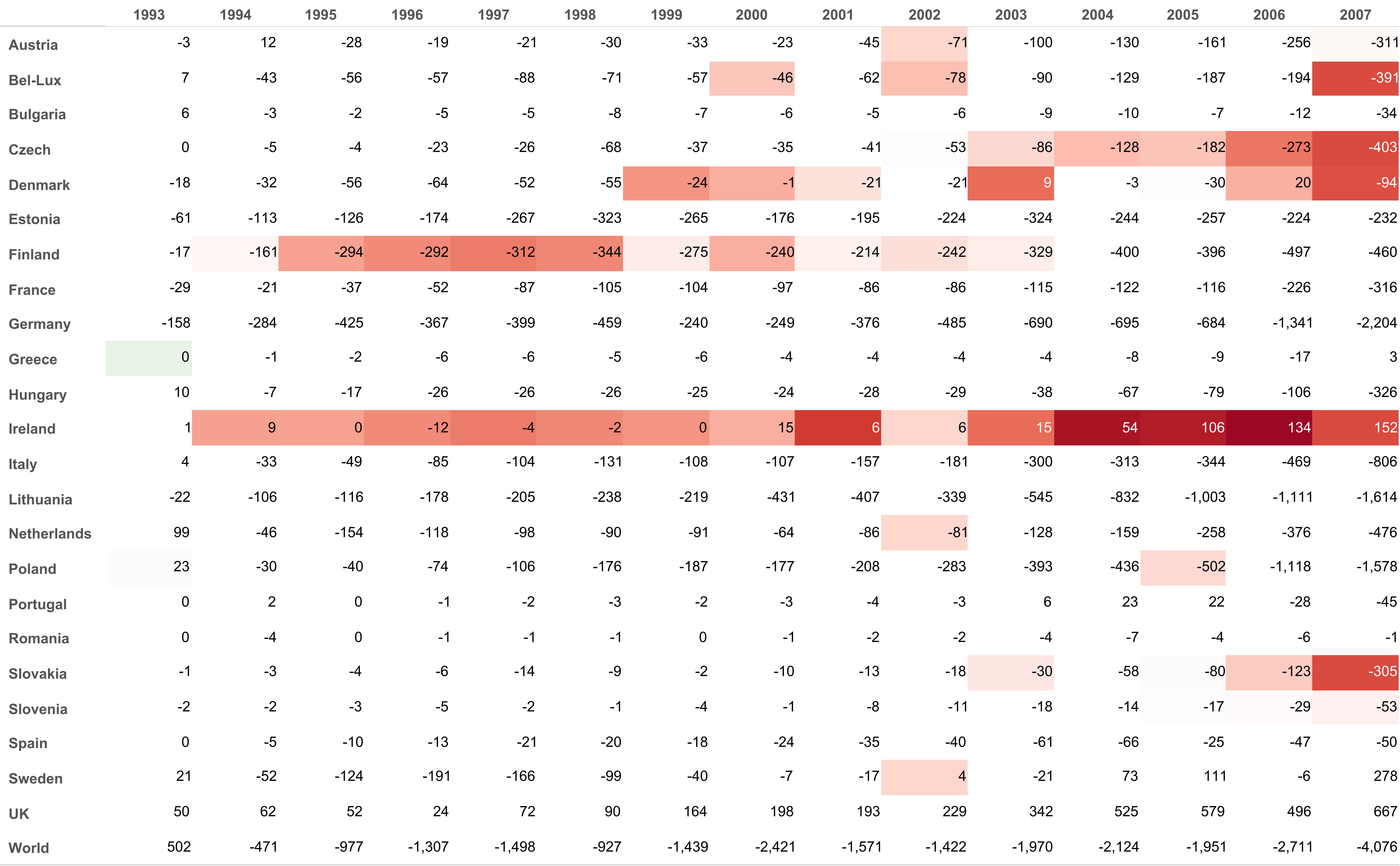

Supplement: Figure S15 — Evolution of the direct and indirect measures of trade imbalances for Latvia. The figures in each cell correspond to direct trade surpluses (+) or deficits (−) of Latvia toward countries listed on the rows. The colors correspond to the indirect measures of trade imbalances, as computed by the Flow Decomposition Method, with ultimate surpluses in green and ultimate deficits in red. (TIFF) [file pone.0083448.s015.tiff]

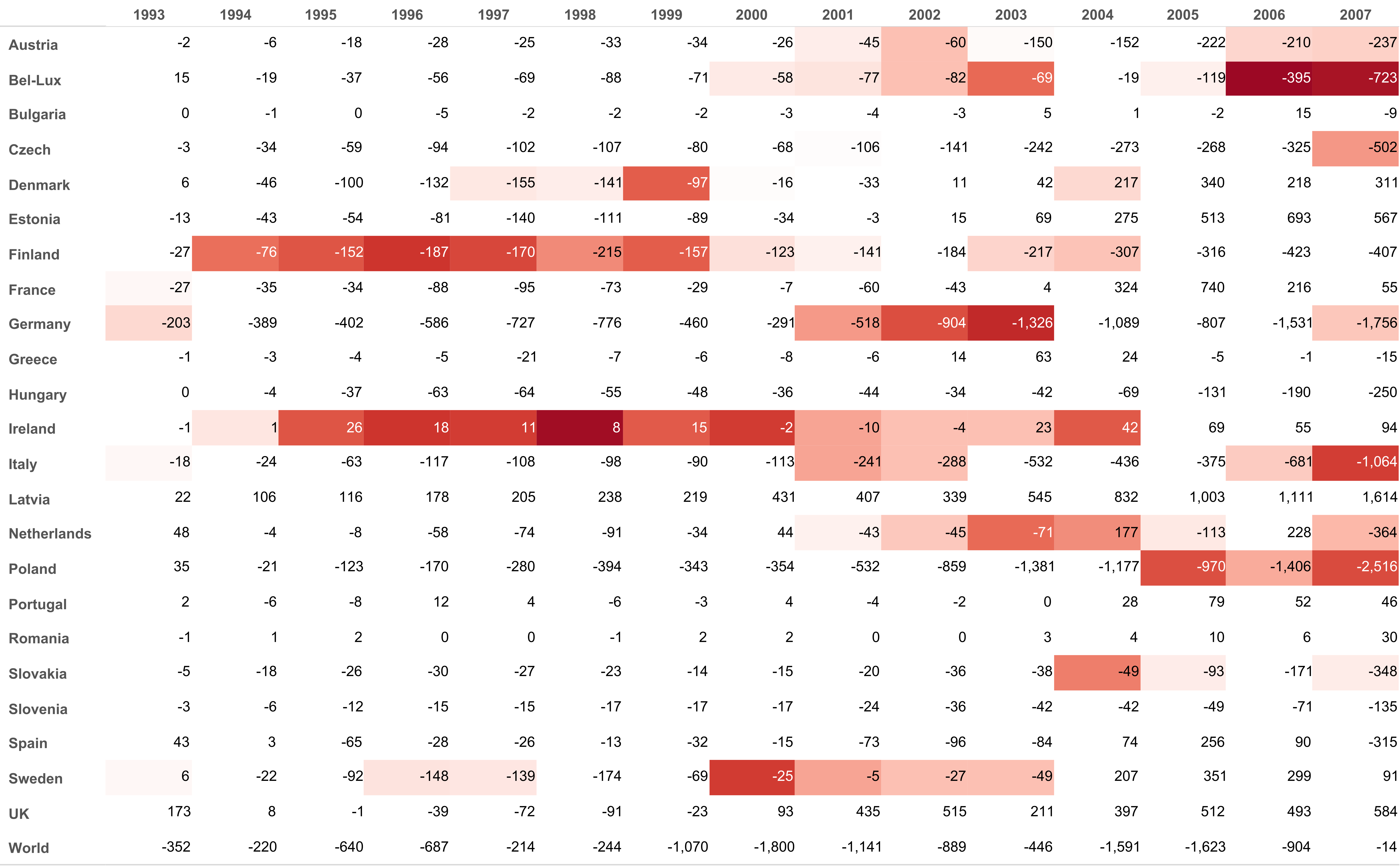

Supplement: Figure S16 — Evolution of the direct and indirect measures of trade imbalances for Lituania. The figures in each cell correspond to direct trade surpluses (+) or deficits (−) of Lituania toward countries listed on the rows. The colors correspond to the indirect measures of trade imbalances, as computed by the Flow Decomposition Method, with ultimate surpluses in green and ultimate deficits in red. (TIFF) [file pone.0083448.s016.tiff]

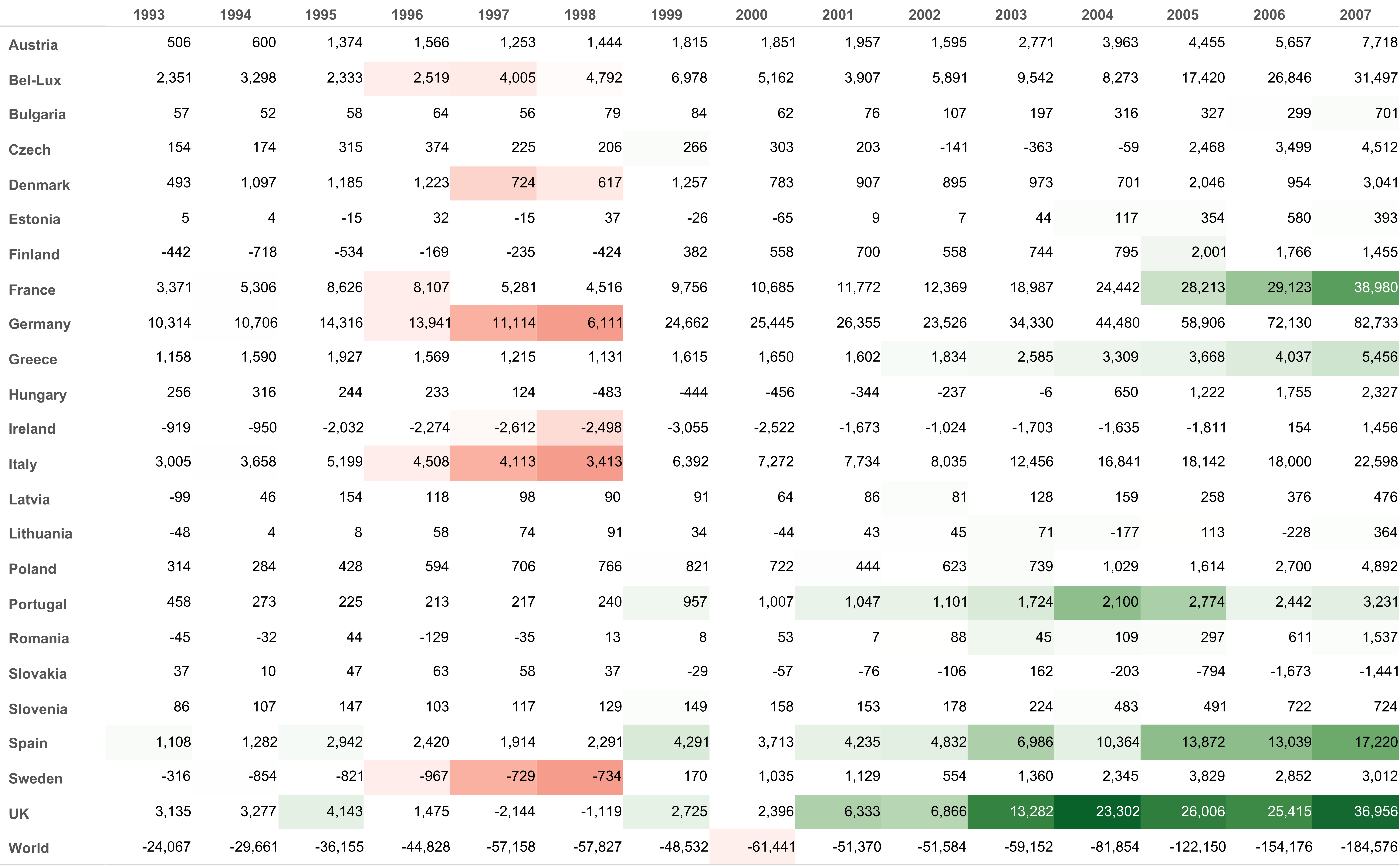

Supplement: Figure S17 — Evolution of the direct and indirect measures of trade imbalances for the Netherlands. The figures in each cell correspond to direct trade surpluses (+) or deficits (−) of the Netherlands toward countries listed on the rows. The colors correspond to the indirect measures of trade imbalances, as computed by the Flow Decomposition Method, with ultimate surpluses in green and ultimate deficits in red. (TIFF) [file pone.0083448.s017.tiff]

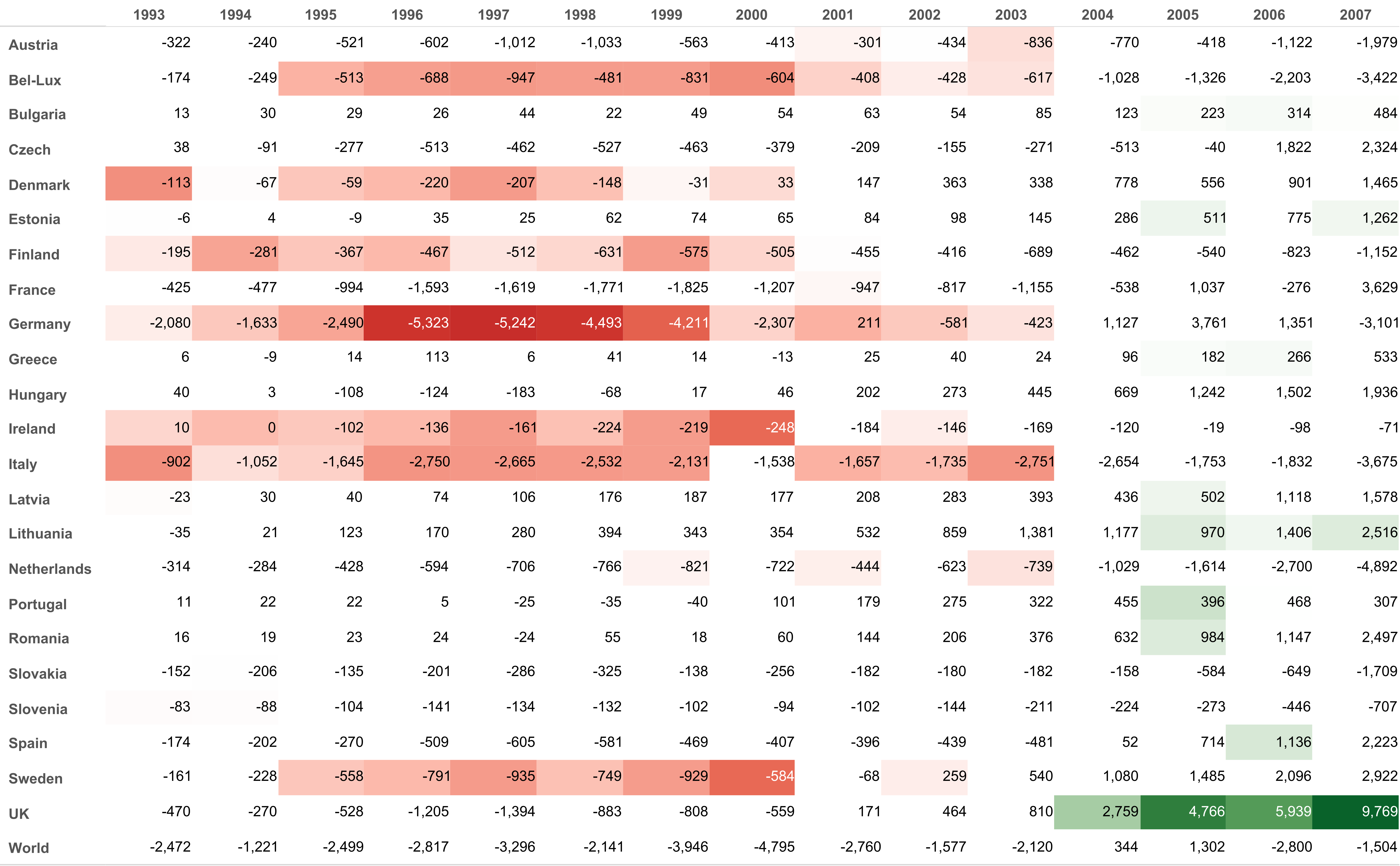

Supplement: Figure S18 — Evolution of the direct and indirect measures of trade imbalances for Poland. The figures in each cell correspond to direct trade surpluses (+) or deficits (−) of Poland toward countries listed on the rows. The colors correspond to the indirect measures of trade imbalances, as computed by the Flow Decomposition Method, with ultimate surpluses in green and ultimate deficits in red. (TIFF) [file pone.0083448.s018.tiff]

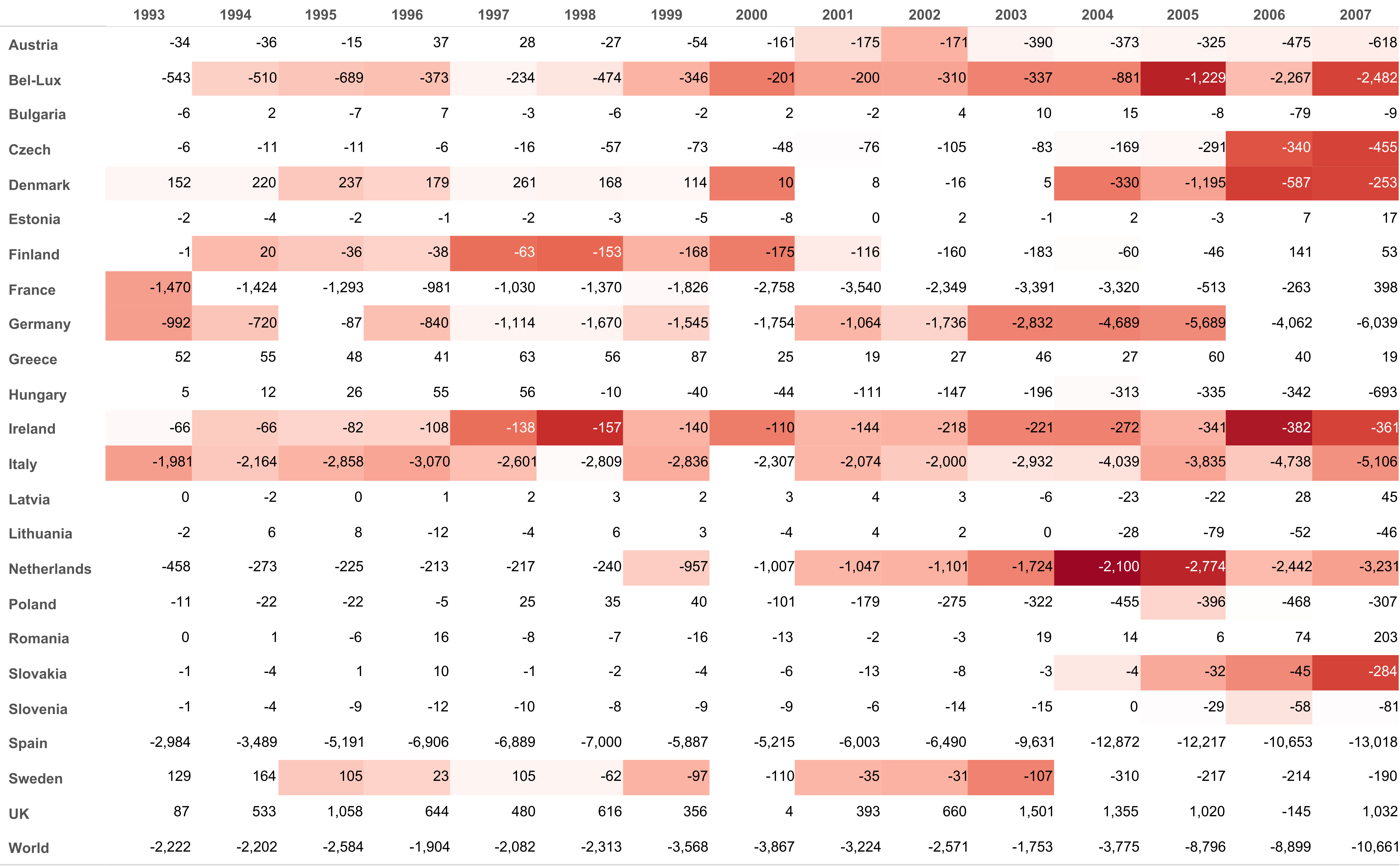

Supplement: Figure S19 — Evolution of the direct and indirect measures of trade imbalances for Portugal. The figures in each cell correspond to direct trade surpluses (+) or deficits (−) of Portugal toward countries listed on the rows. The colors correspond to the indirect measures of trade imbalances, as computed by the Flow Decomposition Method, with ultimate surpluses in green and ultimate deficits in red. (TIFF) [file pone.0083448.s019.tiff]

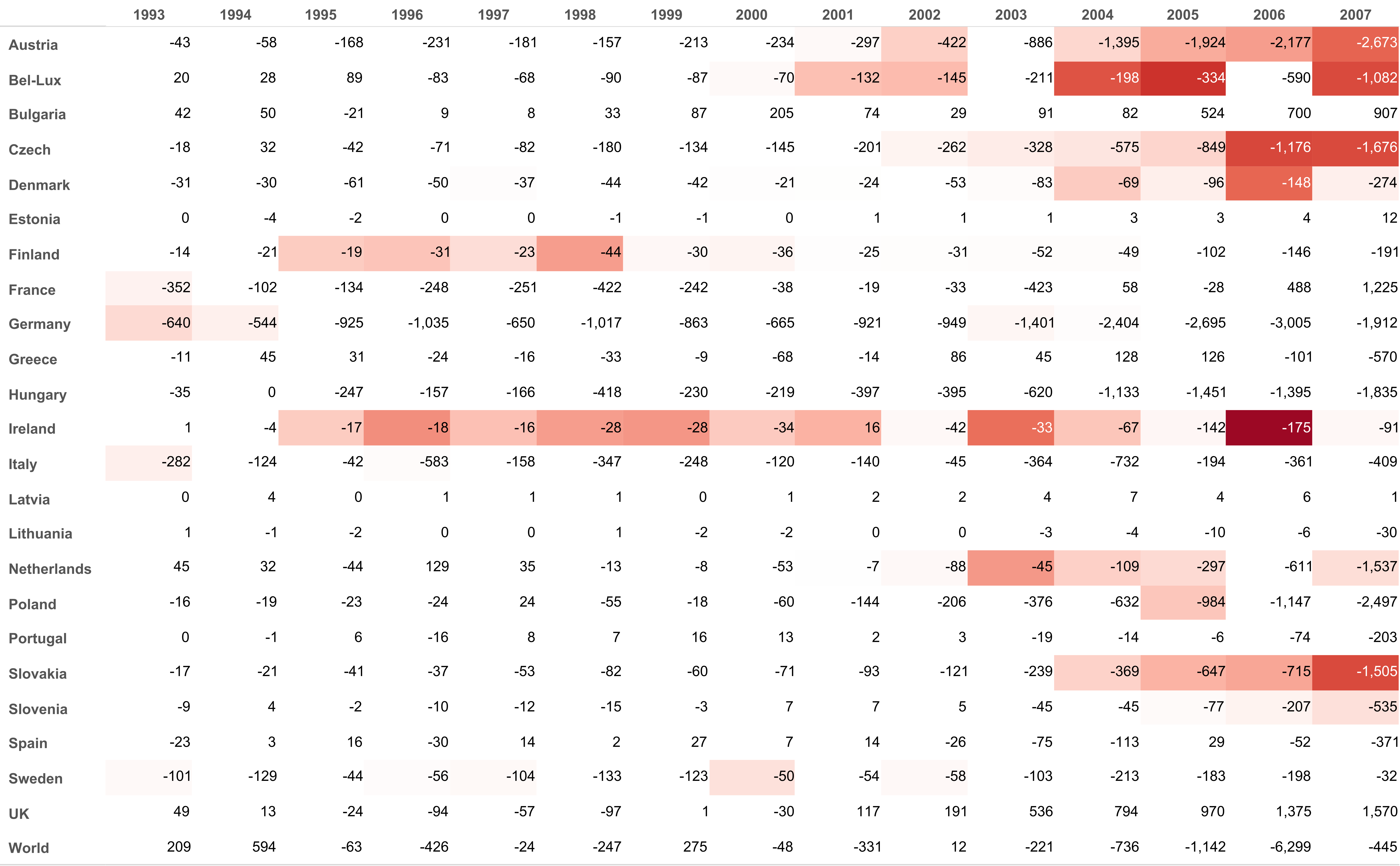

Supplement: Figure S20 — Evolution of the direct and indirect measures of trade imbalances for Romania. The figures in each cell correspond to direct trade surpluses (+) or deficits (−) of Romania toward countries listed on the rows. The colors correspond to the indirect measures of trade imbalances, as computed by the Flow Decomposition Method, with ultimate surpluses in green and ultimate deficits in red. (TIFF) [file pone.0083448.s020.tiff]

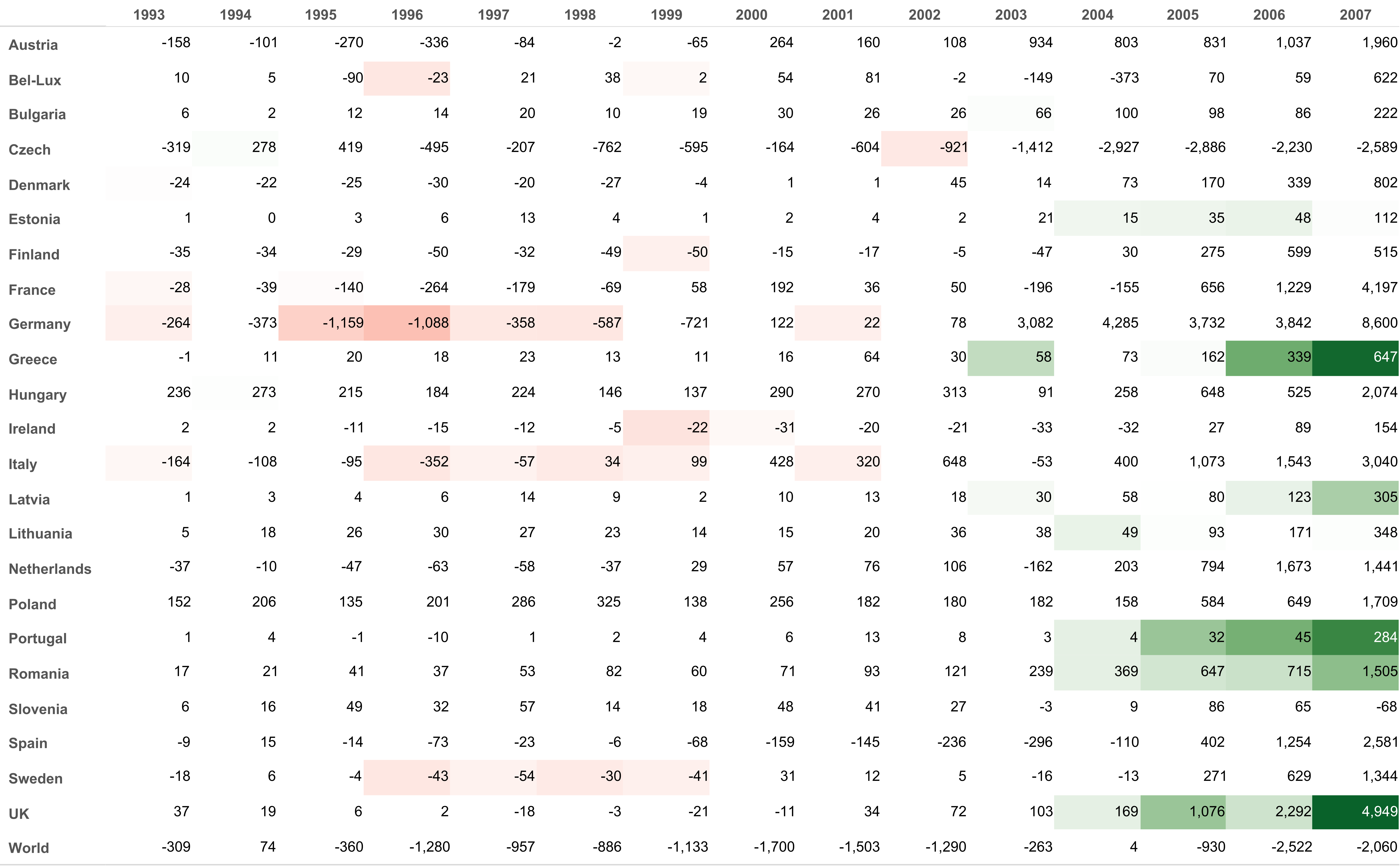

Supplement: Figure S21 — Evolution of the direct and indirect measures of trade imbalances for Slovakia. The figures in each cell correspond to direct trade surpluses (+) or deficits (−) of Slovakia toward countries listed on the rows. The colors correspond to the indirect measures of trade imbalances, as computed by the Flow Decomposition Method, with ultimate surpluses in green and ultimate deficits in red. (TIFF) [file pone.0083448.s021.tiff]

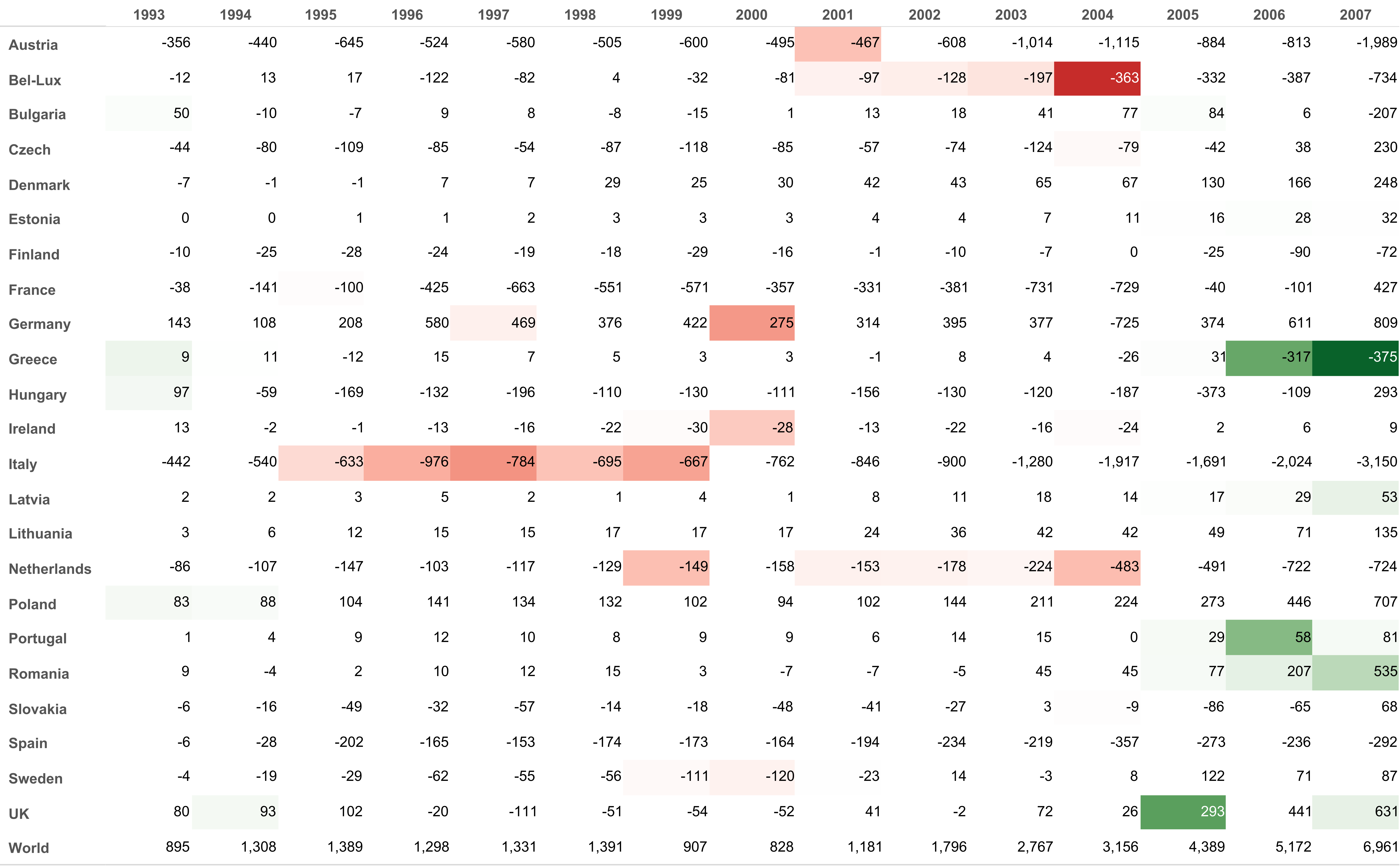

Supplement: Figure S22 — Evolution of the direct and indirect measures of trade imbalances for Slovenia. The figures in each cell correspond to direct trade surpluses (+) or deficits (−) of Slovenia toward countries listed on the rows. The colors correspond to the indirect measures of trade imbalances, as computed by the Flow Decomposition Method, with ultimate surpluses in green and ultimate deficits in red. (TIFF) [file pone.0083448.s022.tiff]

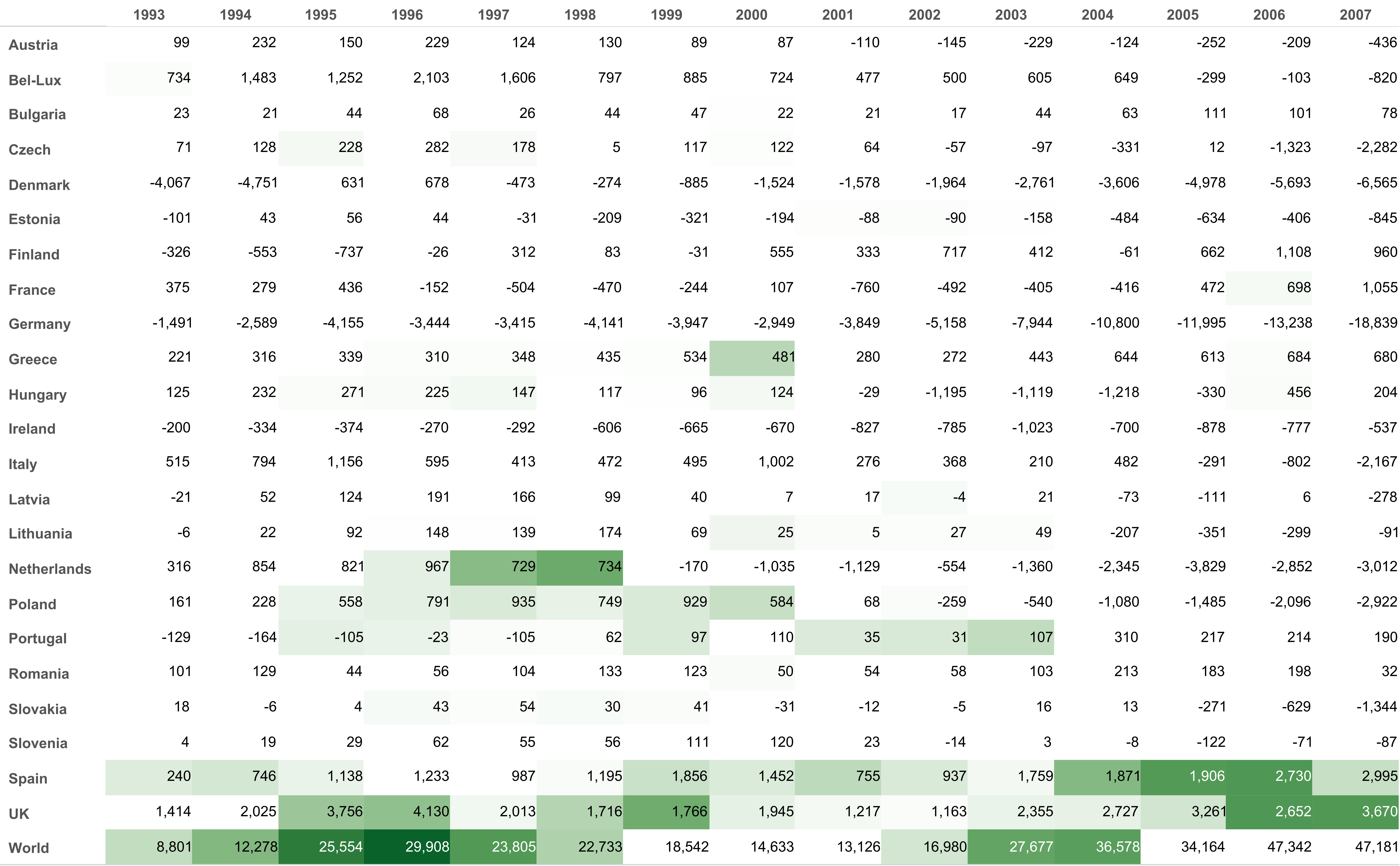

Supplement: Figure S23 — Evolution of the direct and indirect measures of trade imbalances for Sweden. The figures in each cell correspond to direct trade surpluses (+) or deficits (−) of Sweden toward countries listed on the rows. The colors correspond to the indirect measures of trade imbalances, as computed by the Flow Decomposition Method, with ultimate surpluses in green and ultimate deficits in red. (TIFF) [file pone.0083448.s023.tiff]

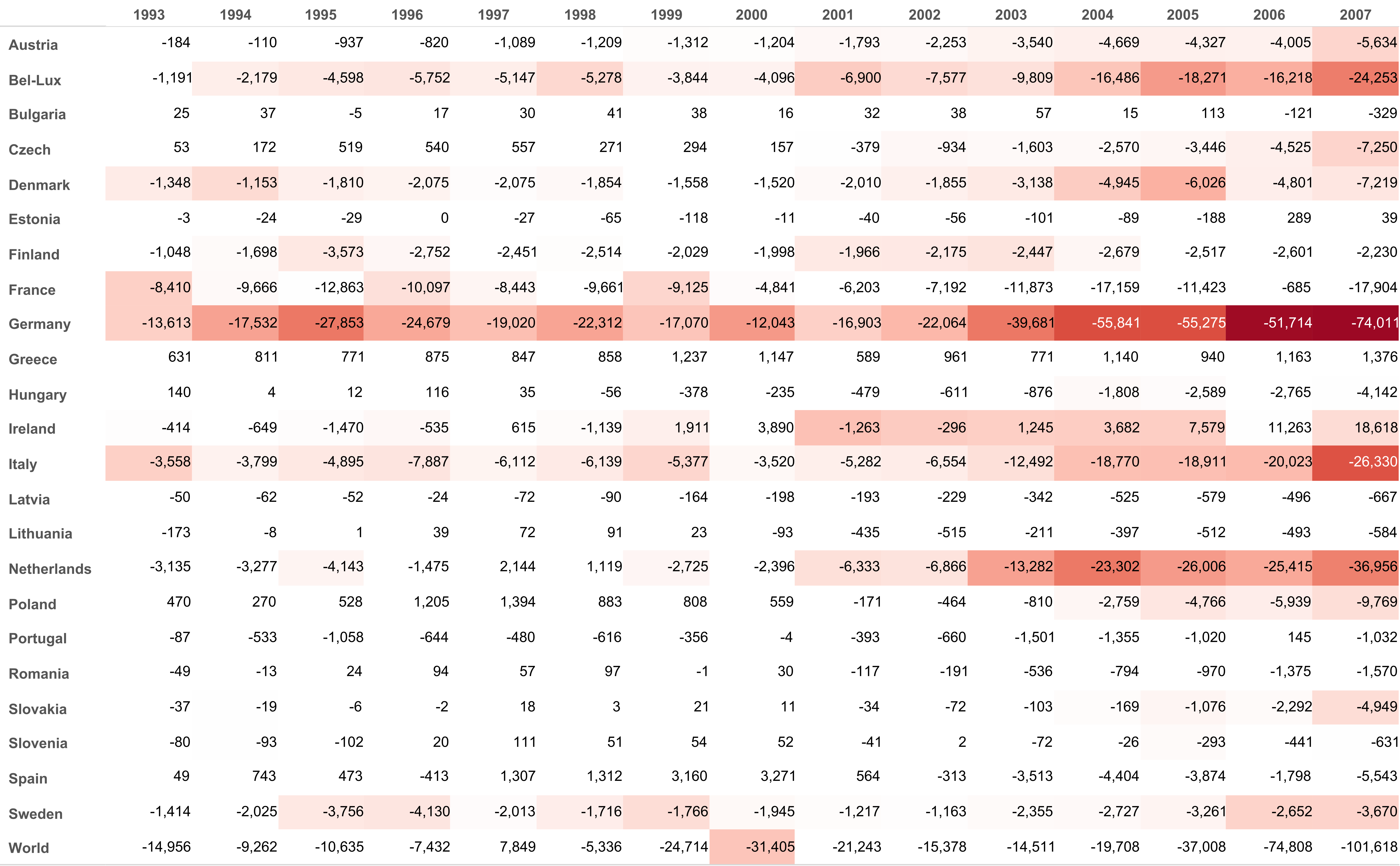

Supplement: Figure S24 — Evolution of the direct and indirect measures of trade imbalances for UK. The figures in each cell correspond to direct trade surpluses (+) or deficits (−) of UK toward countries listed on the rows. The colors correspond to the indirect measures of trade imbalances, as computed by the Flow Decomposition Method, with ultimate surpluses in green and ultimate deficits in red. (TIFF) [file pone.0083448.s024.tiff]
